# Not All Missed Doses Are the Same: Sustained NNRTI Treatment Interruptions Predict HIV Rebound at Low-to-Moderate Adherence Levels

**DOI:** 10.1371/journal.pone.0002783

**Published:** 2008-07-30

**Authors:** Jean-Jacques Parienti, Moupali Das-Douglas, Véronique Massari, David Guzman, Steven G. Deeks, Renaud Verdon, David R. Bangsberg

**Affiliations:** 1 Clinical Research and Biostatistics, and Infectious Diseases Departments, Côte de Nacre University Hospital, Caen, France; 2 Pierre et Marie Curie University Paris 6, UMR S707 and INSERM U707, Paris, France; 3 Epidemiology and Prevention Interventions Center, Division of Infectious Diseases, and The Positive Health Program, San Francisco General Hospital, University of California San Francisco, San Francisco, California, United States of America; 4 Partners AIDS Research Center, Massachusetts General Hospital, Harvard University, Boston, Massachusetts, United States of America; 5 Epidemiology and Prevention Interventions Center, Division of Infectious Diseases, University of California San Francisco, San Francisco, California, United States of America; Institute of Human Virology, United States of America

## Abstract

**Background:**

While the relationship between average adherence to HIV potent antiretroviral therapy is well defined, the relationship between patterns of adherence within adherence strata has not been investigated. We examined medication event monitoring system (MEMS) defined adherence patterns and their relation to subsequent virologic rebound.

**Methods and Results:**

We selected subjects with at least 3-months of previous virologic suppression on a non-nucleoside reverse transcriptase inhibitor (NNRTI)-based regimen from two prospective cohorts in France and North America. We assessed the risk of virologic rebound, defined as HIV RNA of >400 copies/mL according to several MEMS adherence measurements.

Seventy two subjects were studied, five of them experienced virologic rebound. Subjects with and without virologic rebound had similar baseline characteristics including treatment durations, regimen (efavirenz vs nevirapine), and dosing schedule. Each 10% increase in average adherence decreased the risk of virologic rebound (OR = 0.56; 95% confidence interval (CI) [0.37, 0.81], P<0.002). Each additional consecutive day off therapy for the longest treatment interruption (OR = 1.34; 95%CI [1.15, 1.68], P<0.0001) and each additional treatment interruption for more than 2 days (OR = 1.38; 95%CI [1.13, 1.77], P<0.002) increased the risk of virologic rebound. In those with low-to-moderate adherence (i.e. <80%), treatment interruption duration (16.2 days versus 6.1 days in the control group, P<0.02), but not average adherence (53.1% vs 55.9%, respectively, P = 0.65) was significantly associated with virologic rebound.

**Conclusions:**

Sustained treatment interruption may pose a greater risk of virologic rebound on NNRTI therapy than the same number of interspersed missed doses at low-to-moderate adherence.

## Introduction

Adherence to HIV antiretroviral therapy is the strongest predictor of virologic suppression[1], [2], HIV drug resistance[3], disease progression and death[4], [5]. While treatment with unboosted protease inhibitors (PI) requires near perfect adherence for virologic suppression[1], the introduction of more potent non-nucleoside reverse transcriptase inhibitors (NNRTI) and ritonavir boosted PI therapy has lead to reliable virologic suppression at moderate levels of adherence for most, but not all patients[6]–[9].

Because not all patients with moderate adherence are suppressed, we asked if all missed doses are the same. Specifically, we hypothesized that patterns of adherence in addition to average adherence may be an important determinant of incomplete viral suppression. We focused on NNRTI based regimens because these drugs are the cornerstone for most first line regimens world-wide. Because the NNRTIs are potent and have a very long-half life *in vivo*, we hypothesized that once viral suppression was achieved, a sustained treatment interruption rather than frequent missed doses would be associated with virologic failure.

To test this hypothesis, we analyzed prospectively collected data from NNRTI treated individuals who achieved an initial virologic response during adherence monitoring with electronic medication monitors. Electronic medication monitors measure patterns of missed doses with a time-date record of pill bottle opening behaviour. The objective of this study was to identify adherence patterns predictive of virologic rebound on NNRTI-based antiretroviral therapy. We examined the temporal association between average adherence, treatment interruptions and subsequent viral rebound in all subjects, but in particular, subjects with <80% adherence because they are at highest risk for virologic rebound[7], [10].

## Methods

### Patients Population and Study Design

We conducted a case-control study of NNRTI treated patients nested in two prospective observational cohorts: the Posology of Nevirapine (POSOVIR) Study and the Research in Access to Care (REACH) Cohort. POSOVIR is a randomized study of once versus twice daily nevirapine at 4 teaching medical institutions in France[10]. The REACH cohort is an observational study of HIV positive homeless and marginally housed individuals in San Francisco[7]. Written informed consent was obtained from all participants for adherence monitoring, monthly phlebotomy, and assessment of viral load and CD4 cell count. The University of Caen Institutional Review Board (POSOVIR) and the University of California, San Francisco Committee on Human Subjects Research (REACH) approved all study procedures. We selected NNRTI-based treated individuals with HIV RNA level (VL) <400 copies/ml after at least 3 months of treatment and monitored adherence with the AARDEX Medication Event Monitoring System (MEMS). Virologic rebound was defined as VL ≥400 copies/ml at any point during adherence monitoring.

### Adherence Monitoring and Definitions

Adherence was prospectively measured using the Medication Event Monitoring System (MEMS) 6 caps (AARDEX Ltd, Switzerland or Union City, Ca., USA). Individuals with no MEMS events for >15 days immediately prior to virologic rebound were excluded in order to exclude rebound due to simple treatment discontinuation. Percent dose adherence was defined as MEMS events/prescribed number of doses×100. We characterized patterns of missed doses by several a priori measures: (1) number of days without a dose, defined as drug discontinuation for more than 24 hours and less than 48 hours; (2) number of treatment interruptions lasting ≥48 hours, and (3) the duration of the longest treatment interruption (in days).

### Statistical Analysis

Continuous variables were summarized as means, medians, standard deviations and ranges. Dichotomous data were summarized as proportions. Rates were compared by Fisher exact test and continuous variable were compared by *t*-test. The effect of adherence on the probability of virologic rebound was estimated by calculating the exact odds ratios (ORs) and their 95% confidence intervals using univariate exact conditional logistic models, to account for the small sample size with sparse data[11]. Goodness of fit was assessed by the Farrington test for sparse data (GOFLOGIT SAS macro). A logistic regression model curve was used to estimate the relationship between treatment interruption duration and the probability of virologic rebound.

We conducted a sensitivity analysis limited to subjects with <80% adherence in order: (1) to create a closer balance in the distribution of adherence between the groups with and without virologic rebound, since all the subjects in the former group had <80% adherence; and (2) to test whether adherence patterns still predicted viral rebound at low-to-moderate adherence. Quantitative variables were compared between groups with and without virologic rebound by exact Wilcoxon two-sample non-parametric tests because of the uncertainty of whether the small sample met assumptions of a normal distribution.

Analyses were performed using PowerView 2.3.3 (AARDEX Ltd, Switzerland) and SAS version 9.1 (SAS Institute Inc, Cary, NC). All reported *P* values are 2-sided, and *P*<.05 was considered significant.

## Results

### Study population

Seventy-two participants met eligibility criteria. By design, all subjects had a HIV RNA less than 400 copies/ml during at least 3 months of NNRTI-based antiretroviral therapy. Five subjects experienced virologic rebound. Other antiretroviral agents included zidovudine plus lamivudine in 33 (46%), tenofovir plus lamivudine or emtricitabine in 18 (25%), abacavir plus lamivudine in 5 (7%), other nucleosides without protease inhibitors in 11 (15%) and ritonavir-boosted protease Inhibitors in 5 (7%). The mean (SD) duration of MEMS monitoring in days among subjects with and without virologic rebound were 85 (6) and 88 (6) days, respectively. Other baseline characteristics were similar between groups (Table 1).

**Table 1:** Baseline characteristics by study groups.

	Virologic rebound (n = 5)	Virologic control (n = 67)	P value*
Age, y (SD)	47.0 (6,3)	46.8 (10.6)	0.97
Male, No (%)	5 (100)	56 (84)	1.0
Cohort, No (%)			
POSOVIR	2 (40)	50 (75)	0.13
REACH	3 (60)	17 (25)	
Race, No (%)			
Caucasian	4 (75)	61 (91)	0.41
Black	1 (25)	6 (9)	
CD4 cell count, mean (SD)	478 (310)	532 (223)	0.62
CD4 cell nadir, mean (SD)	188 (139)	233 (175)	0.57
Prior exposure to NNRTI in months, mean (SD)	28.4 (22.9)	30,5 (28,0)	0.88
Prior suboptimal nucleoside exposure, No (%)	1 (20)	24 (36)	0.48
Current NNRTI, No (%)			
Nevirapine	4 (80)	56 (84)	1.0
Efavirenz	1 (20)	11 (16)	
Daily dosage, No (%)			
Once-daily	2 (40)	27 (40)	1.0
Twice daily	3 (60)	40 (60)	

* Exact Fisher chi-square test for percentages and *t*-test for continuous variables.

### Predictors of virologic rebound

**Figure 1:**
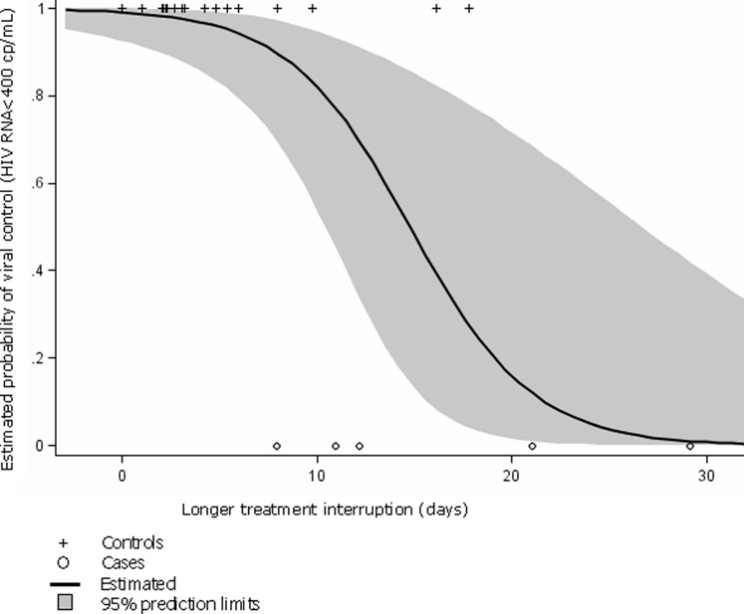
Predicted and observed risk of viral control according to the longer interval of treatment discontinuation, POSOVIR and REACH cohorts.

**Figure 2:**
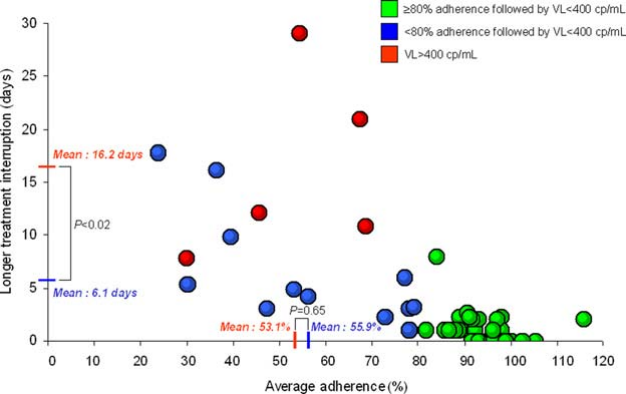
Relationship between average adherence and longer treatment interruption among subjects with (red) and without (green and blue) virologic rebound, POSOVIR and REACH cohorts. - The red lines on the X and Y-axis correspond to mean average adherence (%) and treatment interruption duration (days), respectively, among subjects with subsequent HIV RNA≥400 copies/ml. The blue lines on the X and Y-axis correspond to mean average adherence (%) and treatment interruption duration (days), respectively, among subjects with subsequent HIV RNA<400 copies/ml and low-to-moderate adherence (<80%). The difference in treatment interruption duration, but not adherence rate, is statistically significant between those with HIV RNA≥400 copies/ml and those with HIV RNA<400 copies/ml.

All adherence measurements significantly predicted virologic rebound, except the frequency of short-term interruptions (24 to 48 hours), as shown in Table 2. All explanatory models demonstrated good statistical fit with virologic rebound. The probability of virologic control according to the longer durations of treatment interruption is displayed in Figure 1. Based on our logistic model, a treatment interruption of 15 days was associated with a 50% probability (95% CI = 15%, 86%) of virologic rebound (Figure 1). Figure 2 shows the relationship between average adherence and the longer durations of treatment interruption in days. Not surprisingly, subjects with >80% average adherence also had short treatment interruptions and all of them achieved virologic control.

**Table 2:** Effect of adherence rates and patterns on the risk of virologic rebound.

	Controls (n = 67)	Cases (n = 5)	OR* [95% CI]	P value
Percentage adherence rate§, mean (SD)	88.5 (2.2)	53.1 (7.3)	0.56 [0.37–0.81]	<0.002
No. of days without dose$, mean (SD)	2.1 (0.4)	5.0 (1.4)	1.15 [0.94–1.40]	0.16
No. of TI&, mean (SD)	1.2 (0.3)	8.0 (2.7)	1.38 [1.13–1.77]	<0.002
Longest interval w/o dose, mean in days (SD)	1.5 (0.4)	16.2 (3.9)	1.34 [1.15–1.68]	<0.0001

* OR [95% CI]: Odds Ratio [95% confidence Interval] computed by conditional exact logistic regression. OR>1 means an increased probability of viral rebound.

§ OR and 95% CI are provided for a 10% increase in adherence rate.

$ Days without dose defined as drug discontinuations for more than 24 hours and less than 48 hours.

& TI: Treatment interruptions defined as drug discontinuations for more than 48 hours.

### Sensitivity analyses

In the sensitivity analysis limited to subjects with low-to-moderate (<80%) adherence, the mean difference in longest interruption between those with and without viral rebound was 10.1 days (16.2 vs 6.1, respectively) and statistically significant (p<0.02); whereas, the mean difference in adherence between those with and without viral rebound was 2.8% (53.1% vs 55.9%, respectively) and was not statistically significant (p = 0.65). (Figure 2). There were no other differences in patterns of adherence between the two groups (data not shown).

## Discussion

These data suggest that sustained and repeated NNRTI treatment interruptions are associated with viral rebound. Specifically, sustained treatment interruptions more closely predicted viral rebound than interspersed missed doses in patients with low-to-moderate adherence, which represented 24% of our sample. Average adherence and the potential duration of treatment interruptions are, of course, not independent; 100% adherence precludes an interruption in treatment. As adherence rates decline, however, different patterns of missed doses are possible, as shown in Figure 2. Missed doses can either occur as sustained interruptions or more regularly interspersed missed doses. Our data suggest that not all missed doses are the same. In particular, sustained interruptions of NNRTI-based antiretroviral therapy are more closely associated with viral rebound than the same number of regularly interspersed missed doses among individuals with <80% adherence.

Our finding derived from “real life” treatment interruptions in socio-economically and ethnically diverse patients is consistent with both observational and experimental studies of treatment interruptions. For example, Oyugi et al. found that MEMS defined treatment interruptions with a mean of 11.5 days were associated with NNRTI resistance in Uganda[12]. In two trials, Dybul and colleagues found that repeated long cycles of 4-week off 8-week-on of efavirenz-based therapy was associated with resistance failure[13] while repeated short cycles of 1-week off 1-week-on was not associated with failure[14], even though the 4–8 week schedule delivered more medication over time than that 1–1 week schedule (67% vs 50%, respectively). Similarly, Cohen et al. found that virologic rebound was uncommon among an NNRTI-treated patients with repeated short cycles of 5-days on and 2-days off [15]. Our estimate of 50% probability of viral rebound occurring at 15 days is consistent with these data as well as pharmacokinetic data indicating that efavirenz clearance leads to drug levels that allow for viral replication in 5.8 to 14 days after discontinuation [16].

There are several limitations to our study. The number of events (five) is small, mainly because MEMS technology is not routinely used and because the risk of virologic rebound subsequent to viral suppression on NNRTI-based antiretroviral therapy is low[17]. Larger studies will be needed to confirm our results. Our observational design does not demonstrate causality and we did not measure drug-resistance. However, the risk of resistance after rebound on NNRTI-based therapy is predictably high [13], [18]–[20]. In contrast to structured treatment interruption trials, our study represents the distribution of “unstructured” interruptions common in routine practice. Finally, the result may have been different in a fully antiretroviral naïve population with ongoing viral replication.

Gross et al. found that patients treated with efavirenz-based regimens had suboptimal adherence up to 90 days prior to viral rebound[21]. Our data suggest that patterns of incomplete adherence, namely interruptions in treatment, may narrow this window. In particular, a treatment interruption of 15 days conferred a 50% probability of virologic rebound (Figure 1). Moreover, any treatment interruption of >7 days had a sensitivity of 100% and a specificity of 94% to detect subsequent viral rebound (Figure 2). Thus, the window of opportunity to intervene on risky adherence may depend not only on the level but pattern of adherence.

In summary, near perfect adherence leading to sustained virologic suppression remains the goal of HIV therapy. However, patients with moderate adherence to NNRTIs-based regimens can still achieve virologic control. For these patients with incomplete adherence, missing doses over a continuous and sustained interval may pose more risk for virologic rebound than interspersed missed doses. Limiting sustained NNRTI treatment interruptions may improve durable virologic suppression, especially in patients with incomplete adherence.
